# Supplementary material for: Deconstruction of a Memory Engram Reveals Distinct Ensembles Recruited at Learning
Source: Res Sq. 2025 Feb 5:rs.3.rs-5633532. Preprint. [Version 1] doi: 10.21203/rs.3.rs-5633532/v1 (PMC11838775; doi:10.21203/rs.3.rs-5633532/v1)
Supplement: 1 [file NIHPPrs5633532v1-supplement-1.pdf]

SUPPLEMENTARY MATERIAL

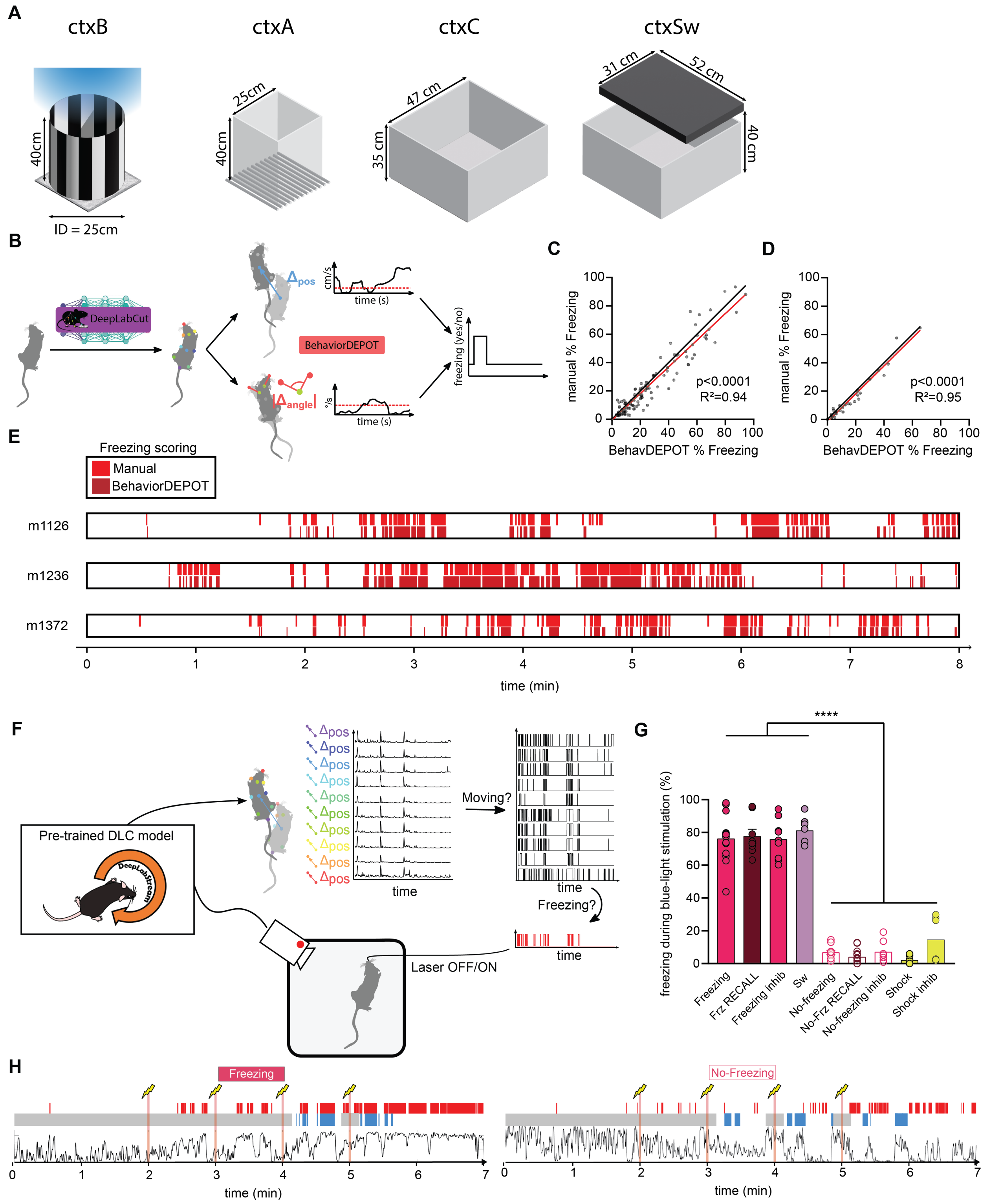

Fig.S1. Offline and live automatic freezing scoring methods. (**A**) Schematics of the different contexts used. (**B**) DeepLabCut and BehaviorDEPOT pipeline for automatic freezing scoring. (**C**) Comparison of automatic and manual freezing scoring with videos split in 20s bouts or (**D**) in 2-minute bouts (simple linear regressions). (**E**) Example animal freezing scores during ctxC using manual versus behaviorDEPOT scoring. (**F**) DeepLabStream pipeline for freezing/no-freezing detection dependent closed-loop tagging. (**G**) Percentage of freezing during blue light tagging for the different groups. (**H**) Example of animals' post-hoc freezing scoring and blue light tagging during FC ctxA. Each data point corresponds to the mean value for an individual animal while bars represent mean ± SEM across animals. ****$p<0.0001$.

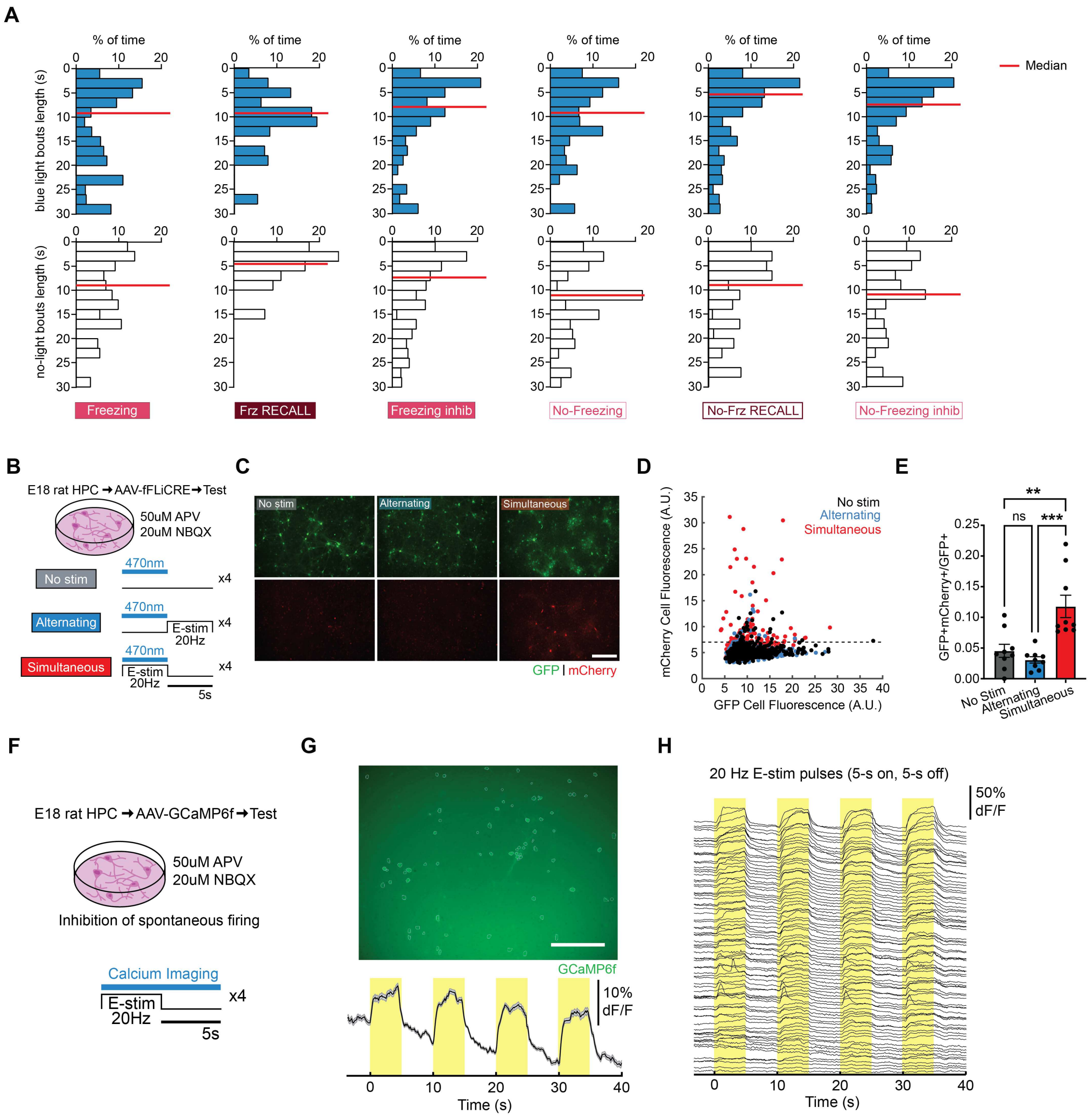

Fig.S2. In-vitro validation of the novel f-FLiCRE tool. (**A**) Weighted histograms representing proportionality of blue-light bouts (blue) or no-light bouts (white) for freezing/no-freezing groups. Median in red. (**B**) Schematic of the protocol for in-vitro f-FLiCRE validation. Neurons expressing f-FLiCRE proteins have their spontaneous firing inhibited with 50 μM APV and 20 μM NBQX, and are stimulated with an electric field at 20Hz for 5 seconds at a time. (**C**) Example FOVs for all three experimental conditions. Scale bar: 100 μm. (**D**) mCherry vs. GFP cell fluorescence from segmented cells. Dotted line represents threshold for positive counting. (**E**) Fraction of GFP+mCherry+/GFP+ cells for the three conditions. (**F**) Schematic of the protocol for in-vitro stimulation/inhibition validation. (**G**) Top: FOV of cultured rat hippocampal neurons expressing AAV5-hSyn-GCaMP6f and treated with electric field stimulation for 5-s on, 5-s off, for a total duration of 40s (5-ms pulse width, 20 Hz pulse frequency). Neurons were treated with 50 μM APV and 20 μM NBQX. Scale bar, 100μm. Bottom: Mean dF/F traces of n = 78 neurons detected in the FOV. (**H**) Individual traces of the responses of all 78 neuron GCaMP6f to the electric field stimulation. Each data point corresponds to the mean value of an individual FOV, while bars represent mean ± SEM across FOVs. Statistical test is an ordinary one-way ANOVA and Tukey's multiple comparisons test. **p<0.01, ***p<0.001.

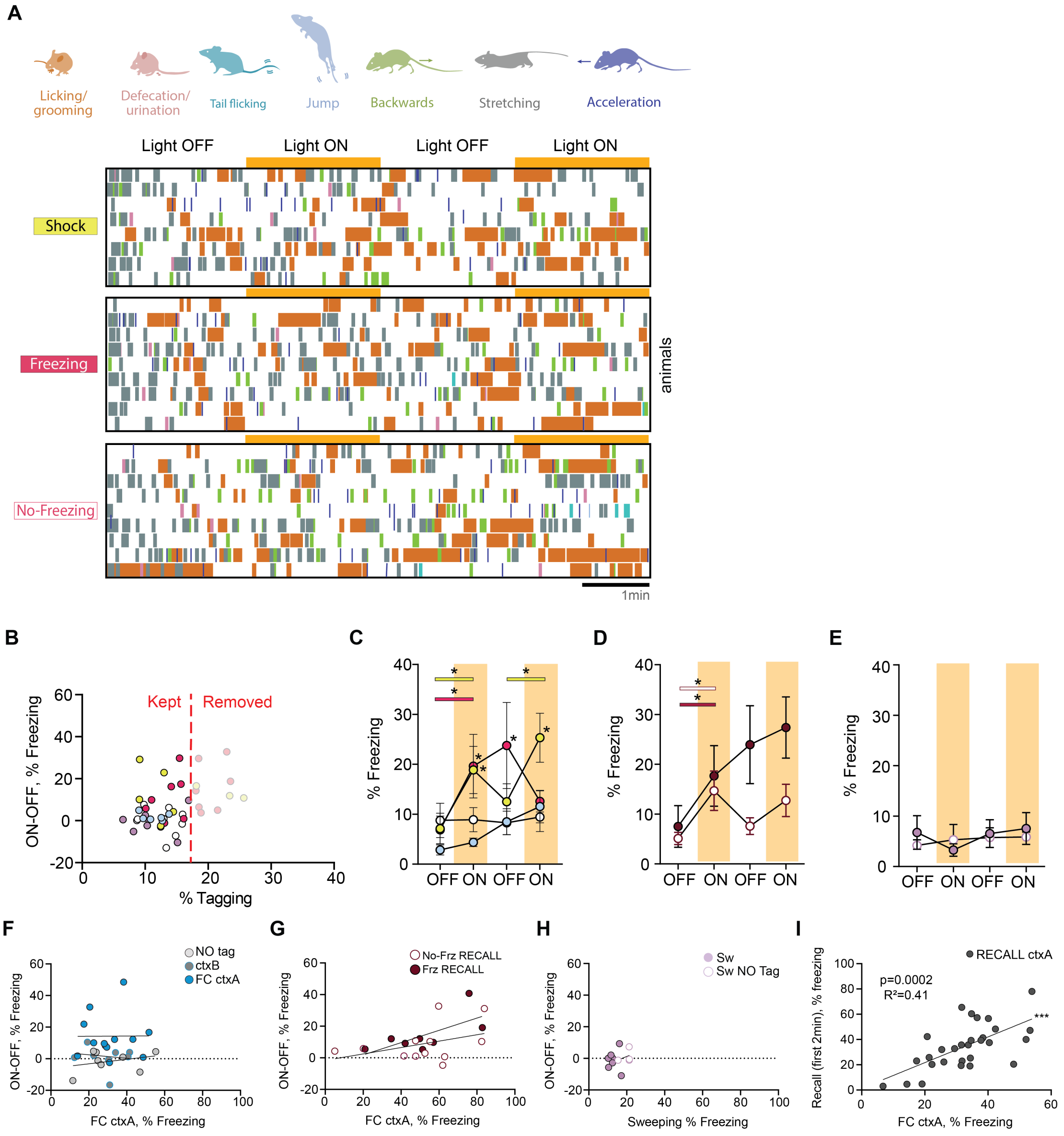

Fig.S3. Additional behavioral tracking and counting. (**A**) Tracking of various behaviors during ctxC in some example groups and animals (RM one-way ANOVA for each type of behavior is non-significant). (**B**) Representation of cutoff % tagged value used to compare the behaviors of animals with similar % tagging values. Comparison of freezing levels between equivalently-tagged animals during light-OFF (no shading) and light-ON epochs (yellow shading) in (**C**) FC ctxA-tagged animals, (**D**) RECALL ctxA-tagged animals, and (**E**) sweeping-tagged animals. (**F**) Correlation between Δfreezing in ctxC and overall freezing in FC ctxA for Fig.1 groups and (**G**) Fig.2P-Q groups (simple linear regressions). (**H**) Correlation between Δfreezing in sweeping-test and overall freezing during sweeping for Fig.2R-S groups (simple linear regressions). (**I**) Correlation between freezing in the first 2 minutes of recall (i.e. before any optogenetic manipulation) and freezing in FC ctxA for all inhibitory groups. Statistical differences between groups are depicted with simple asterisks, while between epochs with color-coded lines, only for consecutive periods (non-consecutive significance not shown). Each data point corresponds to the mean value for each individual animal while bars represent mean ± SEM across animals. *p<0.05, ***p<0.001.

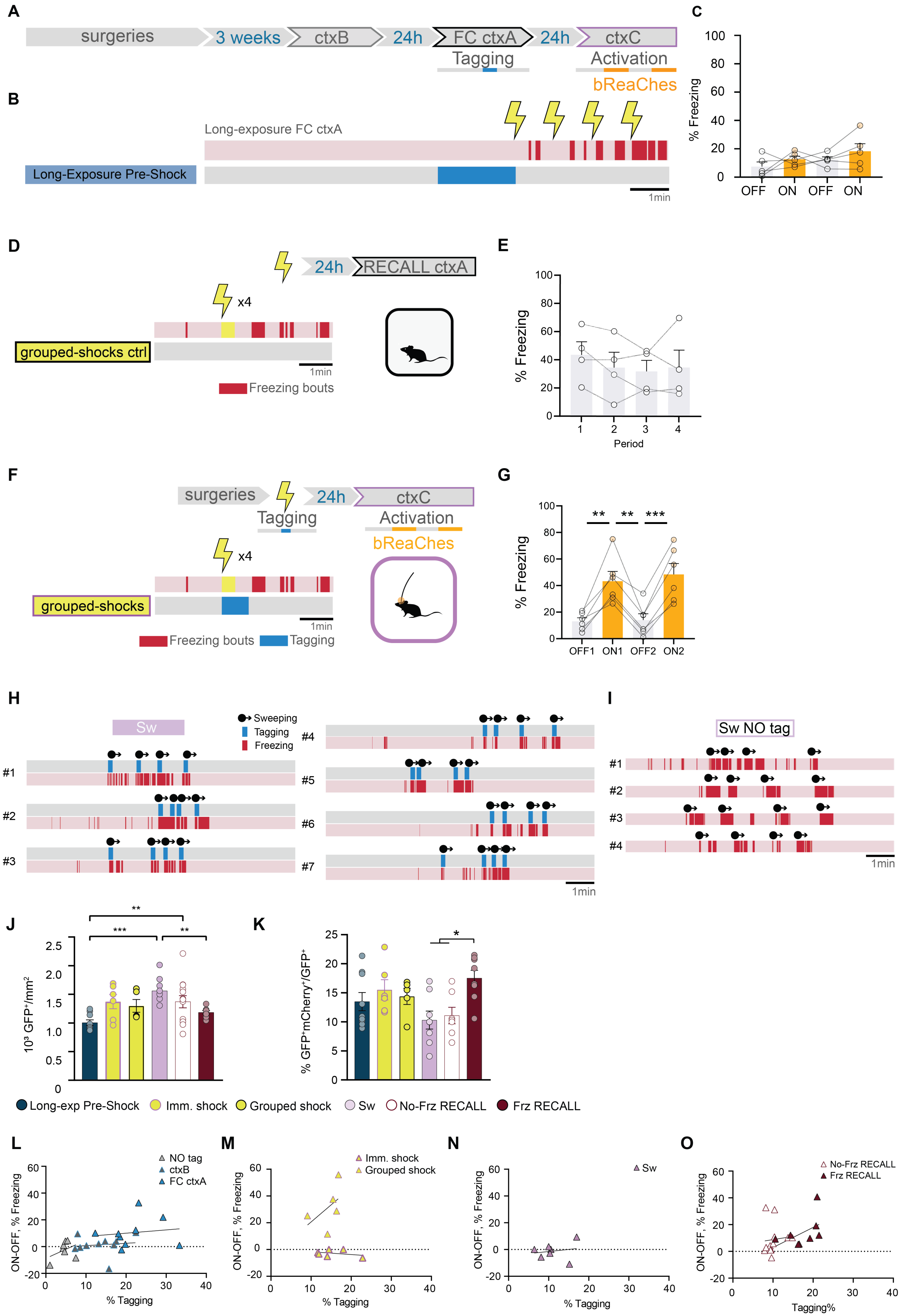
A
surgeries
3 weeks
ctxB
24h
FC ctxA
24h
ctxC
Tagging
Activation
bReaChes
B
Long-exposure FC ctxA
Long-Exposure Pre-Shock
1min
C
% Freezing
OFF
ON
OFF
ON
D
24h
RECALL ctxA
x4
grouped-shocks ctrl
1min
Freezing bouts
E
% Freezing
Period
F
surgeries
24h
ctxC
Tagging
Activation
bReaChes
x4
grouped-shocks
1min
Freezing bouts
Tagging
G
% Freezing
OFF1
ON1
OFF2
ON2
H
Sw
Sweeping
Tagging
Freezing
1min
I
Sw NO tag
1min
J
10^3 GFP+/mm2
K
% GFP+mCherry+/GFP+
Long-exp Pre-Shock
Imm. shock
Grouped shock
Sw
No-Frz RECALL
Frz RECALL
L
NO tag
ctxB
FC ctxA
ON-OFF, % Freezing
% Tagging
M
Imm. shock
Grouped shock
ON-OFF, % Freezing
% Tagging
N
Sw
ON-OFF, % Freezing
% Tagging
O
No-Frz RECALL
Frz RECALL
ON-OFF, % Freezing
Tagging%

Fig.S4. Long exposure, sweeping, and RECALL tagging experiments. (**A**) Long-exposure pre-shock experiment protocol and (**B**) tagging timeline. (**C**) Freezing levels of individual "long-exposure pre-shock" animals during ctxC, outside (gray) and during (yellow) light-on periods. (**D**) Schematic of grouped-shock ctrl experiment: After training in ctxA with the same shock patterns as immediate shock groups, freezing is tested in a recall ctxA session. (**E**) Freezing level in recall ctxA. (**F**) Schematic of grouped-shock experiment: after opto-tagging in ctxA with the same shock patterns as immediate shock groups, freezing is tested in an opto-reactivation test ctxC session.(**G**) Freezing level in test ctxC. (**H**) Timelines of all "Sw" and (**I**) "Sw NO tag" animals, displaying sweeping bouts, freezing, and tagging for the former. (**J**) Density of GFP+ (i.e. infected) neurons in the dCA1. (**K**) Percentage of tagged cells (GFP+mCherry+/GFP+). (**L**) Correlation between Δfreezing in ctxC/sweeping and % of tagged cells for Fig.1 groups. (**M**) Correlation between Δfreezing in ctxC and % of tagged cells for imm.shock and grouped shock groups (**N**) Correlation between Δfreezing in sweeping-test and % of tagged cells for "Sw" tagged animals. (**O**) Correlation between Δfreezing in ctxC/sweeping and % of tagged cells for Fig.2K-L groups. Each data point corresponds to the mean value for each individual animal while bars represent mean ± SEM across animals. $^{**}p<0.01$, $^{***}p<0.001$.

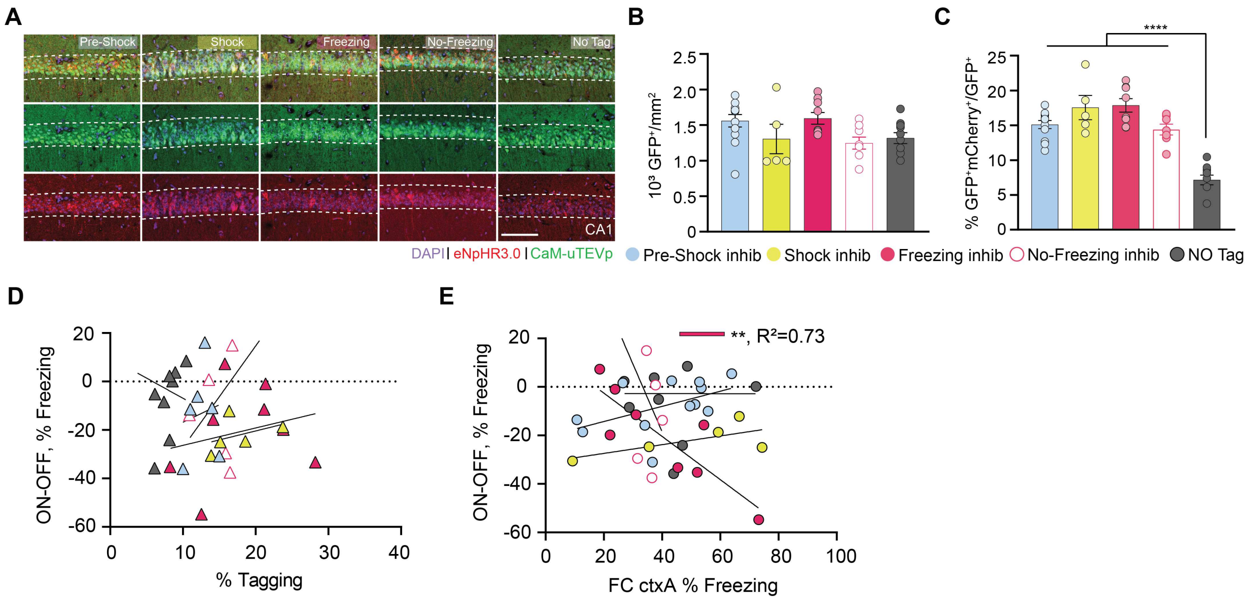

Fig.S5. FLiCRE-tagging and behavior analysis in inhibitory groups. (**A**) Representative images of infection (green) and tagging (red) for inhibitory groups. Scale bar: 100µm. (**B**) Density of GFP+ (i.e., infected) neurons in the gCA1 for the inhibitory groups. (**C**) Percentage of tagged cells (GFP+mCherry+/GFP+). (**D**) Correlation between Δfreezing in RECALL ctxA and % of tagged cells for inhibitory groups. (**E**) Correlation between Δfreezing in RECALL ctxA and overall freezing in FC ctxA for inhibitory groups. Each data point corresponds to the mean value for an individual animal while bars represent mean ± SEM across animals. Statistical tests are ordinary one-way or linear regressions depending on context. **p<0.01, ****p<0.0001.

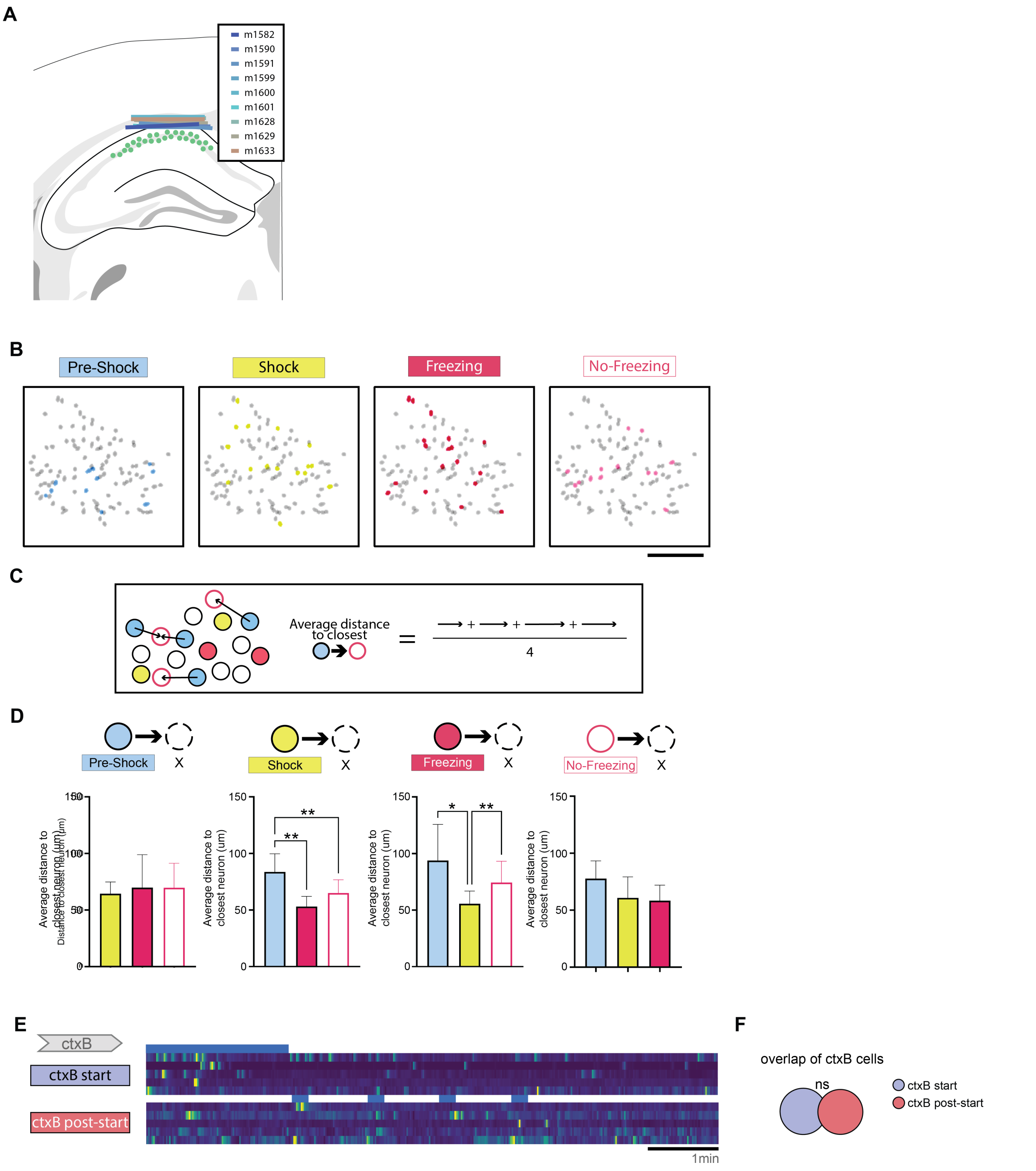

Fig.S6. Calcium imaging: validation, overlap statistics, and topological analysis. (**A**) Schematic of lens positioning and infection for all 9 animals implanted for calcium imaging experiments. (**B**) Representative animal's miniscope field of view, with the topology of the 4 tracked 'FLiCRE-like' groups shown. Scale bar: 200µm. (**C**) Computation of average distance to closest neighbor between two cell groups. (**D**) Average distance to closest neighbor between all pairs of cell groups (ordinary one-way ANOVA). (**E**) Example calcium traces from ctxB. Blue represents the periods used to determine which cells belong to the corresponding group, and would have been tagged using FLiCRE. (**F**) Overlap between cell groups of ctxB. Each data point corresponds to the mean value for an individual animal while bars represent mean ± SEM across animals. *p<0.05, **p<0.01.

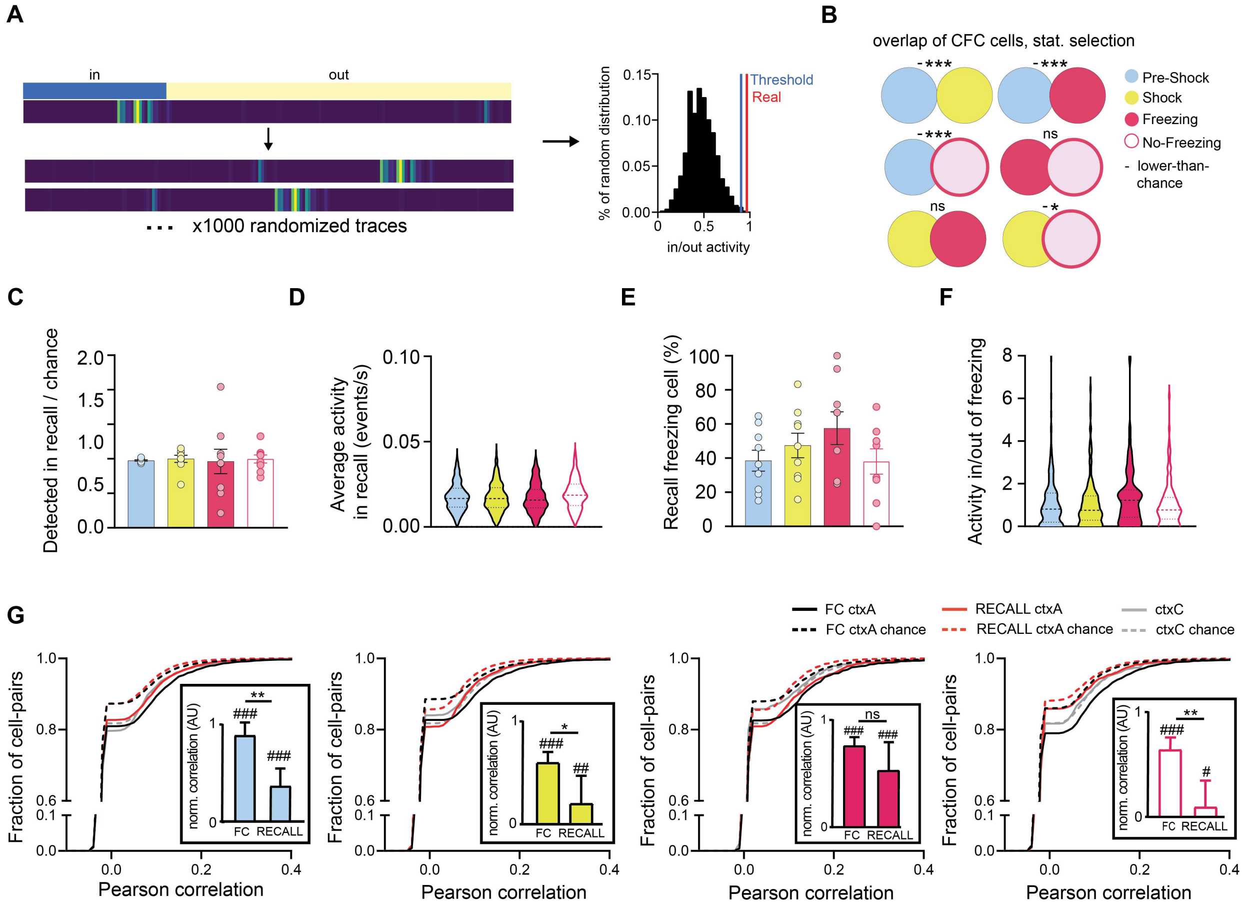

Fig.S7. Calcium imaging: Analysis with statistical criteria. (**A**) Schematic of method for sorting cells into the "Pre-Shock", "Shock", "Freezing", and "No-Freezing" groups using a statistical criterion (example shown for pre-shock). (**B**) Overlap between cell groups from the same experiment, top: FC, and bottom: recall. + denotes higher-than-chance overlaps, - lower than chance overlaps. (**C**) Number of cells per animal from FC ctxA cell groups detected in RECALL ctxA over chance number by cell group. (**D**) Average calcium events per second in RECALL ctxA for tracked cells by cell group. (**E**) Percentage of FC ctxA groups cells that are freezing cells in Test ctxA. (**F**) Activity ratios per animal of FC ctxA groups for cells inside/outside of Test ctxA freezing bouts. (**G**) Cumulative distributions of same-group cell-pair Pearson correlations in FC ctxA (black lines), RECALL ctxA (red lines) and ctxC (gray lines). Chance-level correlations are shown with dotted lines. Statistical tests are comparisons to bootstrapped distributions for statistical selection and for overlaps, and ordinary one-way ANOVA otherwise. *p<0.05, ***p<0.001, #p<0.05, ###p<0.001.

Movie S1. Example ctxC session of "Pre-Shock" tagged animal. Excerpt from test session in ctxC, from minute 1 to minute 3 (i.e. one minute light OFF, one minute with light ON), sped up 4 times. Freezing is indicated as scored by BehaviorDEPOT.

Movie S2. Example ctxC session of "Shock" tagged animal. Excerpt from test session in ctxC, from minute 1 to minute 3 (i.e. one minute light OFF, one minute with light ON), sped up 4 times. Freezing is indicated as scored by BehaviorDEPOT.

Movie S3. Example ctxC session of "Freezing" tagged animal. Excerpt from test session in ctxC, from minute 1 to minute 3 (i.e. one minute light OFF, one minute with light ON), sped up 4 times. Freezing is indicated as scored by BehaviorDEPOT.

Movie S4. Example ctxC session of "No-Freezing" tagged animal. Excerpt from test session in ctxC, from minute 1 to minute 3 (i.e. one minute light OFF, one minute with light ON), sped up 4 times. Freezing is indicated as scored by BehaviorDEPOT.
